# Update of the Impact of Consumption of Whole Chicken Eggs on the Lipid Profile: to What Extent are They Impacting?

**DOI:** 10.5935/abc.20180092

**Published:** 2018-07

**Authors:** Heitor Oliveira Santos

**Affiliations:** Universidade Federal de Uberlândia (UFU), Uberlândia, MG - Brazil

**Keywords:** Cholesterol / chemistry, Eggs / utilization, Egg Proteins, Egg Proteins, Dietary, Antioxidants

## Introduction

The older literature (mid 1980-90) shows that increased dietary cholesterol intake can raise total serum cholesterol and LDL levels.^1,2^ Some more current positions question the recommendation of daily cholesterol intake, as well as the impact of the whole chicken egg, inquiring whether it is a harmful or beneficial classification food in this context.^3,4^ In the midst of controversial issues, it is essential to analyze variables such as food consumption in general and how much dietary cholesterol intake is impacting on the parameters of the lipid profile, thus obtaining a more reliable information, mainly when the focus is the clinical conduct.

One of the foods most known to contain cholesterol is the egg, and it is in the yolk where the cholesterol is concentrated. The chicken egg is the most consumed worldwide, being a food of affordable price, and, above all, of practical cooking and good nutritional profile.^3^

Among the types of cooking, the fried chicken egg, stir-fried, cooked, pochê, roast, in the form of omelet and soufflés, besides being an ingredient of various preparations, stands out. The nutritional aspects of the chicken egg are extensive. It is a source rich in proteins of high biological value, unsaturated fats, liposoluble vitamins (mainly vitamin A and E), vitamin B12 and antioxidant components.^3^

I carried out studies of great sample character, exhibiting interesting adjustments through the ingestion of chicken eggs. Data from observational methodology and human intervention support new research.^5,6^

Hence, we sought to analyze the impact of the intake of whole chicken eggs on the lipid profile from older and recent studies.

### Pre-established studies: discussion of research in the mid-2000s

In 2000, McNamara^7^ through a review encompassing a survey of 167 studies, whereof cholesterol intake in more than 4000 individuals was analyzed, shows that every 100 mg of dietary cholesterol intake the total plasma cholesterol increased only 2.2 mg/dL. A whole chicken egg unit (~ 50 g) contains a cholesterol content equivalent to the amount analyzed in the McNamara study,^7^ presenting approximately 100-150 mg of cholesterol,^8^ therefore, being one of the main foods that provided the increase of the cholesterol intake.^7,8^

Regarding the LDL and HDL lipoproteins, the addition of 100 mg of cholesterol per day from the diet in the general McNamara^7^ population increased the LDL levels by 1.9mg/dL and HDL levels by 0.4 mg/dL. Nevertheless, the mean change in the proportion of LDL: HDL per 100mg of dietary daily cholesterol in the patients was 2.60 to 2.61, that is, both values were literally negligible for the outcome of cardiovascular problems, such as plaque atheroma and stroke.^7^

McNamara^7^ also shows the relationship of dietary cholesterol response to heterogeneity, whose people are divided into hypersensitive and hypersensitive. According to the author, 15% to 25% of the population is sensitive to dietary cholesterol, in which there is a genetic increase in the production of apolipoprotein E4 and apolipoprotein B. However, the impact of daily dietary cholesterol intake per 100mg increased by only 1.4 mg/dL of total cholesterol levels in hypo-responsive individuals and 3.9 mg/dL in hyperresponsive individuals.^7^

However, McNamara^7^ does not emphasize whether the main food responsible for dietary cholesterol was chicken egg. A subject to analyze is the response of the lipid profile of patients with dyslipidemia to the eggs ingestion. In 1997, Knopp et al.^9^ already had data for this issue.

Knopp et al.^9^ conducted a very well controlled study involving 130 patients with hypercholesterolemia and combined hyperlipidemia. For six weeks was used a diet based on traditional recommendations, and from that period randomized patients to receive two whole chicken eggs per week or at most one whole chicken egg per week, lasting 12 weeks. They analyzed the lipid profile before and after the intervention and observed that patients with hypercholesterolemia who consumed two eggs daily increased their HDL levels by an average of 48 to 52 ng/dL (p = 0.003), while they did not change other markers such as LDL, total cholesterol, VLDL and apolipoprotein B. On the other hand, patients with combined hyperlipidaemia who consumed two eggs per day increased mean total cholesterol from 238 to 250 ng/dL (p = 0.001), LDL from 150 to 162 ng/dL (p = 0.001), HDL from 42 to 45 ng/dL (p = 0.02), and apolipoprotein B (p = 0.05). A decrease in VLDL from 103 to 95 mg/dL (p = 0.007) was observed in this study, which was probably not expected.^9^

### Current studies: from large population samples to intervention with good control of intakes of whole chicken eggs

A recently published study showed that consumption between two to four (n = 4493) and greater than four whole chicken eggs (n = 214) per week did not increase the incidence of cardiovascular disease compared to individuals with a consumption habit of less than two eggs per week (n = 2509). It is noteworthy that this study is of the renowned group PREDIMED (PREvención con DIeta MEDiterránea), in which the individuals followed a Mediterranean food style, therefore, with habitual consumption of olive oil and oilseeds. In this way, the increase in egg consumption was accompanied by a good dietary style, which was confirmed by the control of macronutrients and type of ingested lipids, as a relevant percentage of monounsaturated fats (~20% of total caloric value) and moderate in saturated fats (~10% of the total caloric value).^5^

Despite the study by the PREDIMED group,^5^ in a study with a considerable sample (n = 1231) it is stated that the higher the consumption of whole chicken eggs, the greater the area of the atheroma plaque in the carotid (weekly consumption between one and five eggs exhibited larger area of the plaque compared to half egg weekly). However, in the study lack physical exercise control, waist circumference and mainly, dietary habits. Nevertheless, the increase in plaque area was related to an older age and history of smoking.^10^

Another recent study showed that the daily intake of two to three whole chicken eggs increased the functionality of HDL and plasma carotenoids, which are anti-inflammatory and antioxidant factors. Thirty-eight healthy participants participated in a study in which initially they stayed a period of 2 weeks without eating egg, and later consumed an entire egg of chicken for 4 weeks, and progressively two and three whole eggs daily every 4 weeks; the intervention lasted 14 weeks. Compared with the time of deprivation of egg ingestion, consumption of one to three eggs/day resulted in increased concentrations of large LDL (21-37%), large HDL (6-13%) and apolipoprotein AI (9-15%) whereas ingestion of two to three eggs/day promoted an increase in apolipoprotein AII by 11% and lutein and plasma zeaxanthin by 20-31%, whereas the ingestion of three eggs/day resulted in an increase of 9-16% in serum paraoxonase-1 activity compared to the intake of one to two eggs/day. Intake of one egg/day was sufficient to increase HDL function and particle concentration of large LDL. In general, the daily intake of less than three eggs/day favored a better LDL particle profile, improved the function of HDL and increased plasma antioxidants in healthy young adults.^6^

This recent intervention adds to the literature a more biologically detailed impact as a function of the consumption of whole chicken eggs, since it goes beyond classic lipid profile markers in clinical practice, analyzing lipoprotein precursors and antioxidant character.^6^ The increase of paraoxonase-1 and apolipoprotein AI levels as a function of egg ingestion are beneficial, since they are precursors of HDL formation, providing greater functionality.^11,12^ In reference to the increase of large LDL, it does not mean a bad factor, but a beneficial modulation of the molecule, because the higher the volume, the lower is the propensity for endothelial penetration in the arteries, unlike LDL of lower volume (ie, sdLDL, small dense low-density lipoprotein particles).^13^

### Positioning of guidelines regarding cholesterol consumption

The V Brazilian Guideline on Dyslipidemias and Prevention of Atherosclerosis encourages the ingestion of cholesterol < 300 mg/d for patients in general, and for dyslipidemics the incentive is < 200 mg/d.^14^ In agreement with the most recent recommendations in the medical literature, a new consensus from the American Heart Association,^15^ based mainly on the dietary guidelines of the Dietary Guidelines for Americans,^16^ in the period from 2015 to 2020, cholesterol consumption is still limited to that advocated by the V Brazilian Guideline on Dyslipidemias and Prevention of Atherosclerosis.^14^ However, such recommendations do not specify where dietary cholesterol comes from, for example, whether it is primarily from a Western-style diet rich in fritters as a whole, or from a type of nutrient-rich diet rich in functional substances. The American Heart Association's recent positioning encourages Mediterranean feeding, citing the PREDIMED group, which, in parallel, can be based on a considerable weekly frequency of intakes of whole eggs.^15,16^

## Discussion

Analyzing a food in isolation requires detailed adjustments, and the egg is a food that undoubtedly remains controversial. There are two strands, one more cautious and another one that overestimates the potential of the egg as food. Following the traditional recommendations of cholesterol intake is of some importance; however, one should consider the whole lifestyle.

Probably, in individuals who exercise and have good dietary control, the routine intake of whole eggs will not cause harm to the lipid profile, since, presumably, the body is in a good redox balance, being a protective factor for cardiovascular outcomes.^17^

Even in elderly individuals, the daily consumption of eggs, at least non-abusively, may be insignificant in altering the lipid profile.^18^ In a cross-over study of 33 elderly (mean age 79 years), the daily consumption of a whole chicken egg for five weeks did not change any traditional lipid profile marker compared to the same period without the ingestion of eggs, and serum antioxidant markers (+26% lutein and + 38% zeaxanthin).^19^

Given the importance of redox balance as a protector to the cardiovascular side, perhaps the consumption of whole eggs is also not of concern for patients with dyslipidemias, because as was quoted, its consumption exhibits beneficial antioxidant modulation to lipoproteins. The PREDIMED study is a good baseline, encompassing middle-aged and large sample patients. Regarding whole eggs intake in the style of Mediterranean food, two to four eggs per week are consumed on average, whereas less than two servings of sweetmeats and red meat and less than a portion of processed meat are consumed. The white meat intake is two servings and the portions of vegetables, fish or shellfish are two or more per week.^19^ Thus, like the PREDIMED study, if the individual has good eating habits as a whole, the intake of whole eggs with considerable weekly frequency seems to be safe. Above all, the prescription of eggs in clinical practice is a very individual factor, mainly depending on lipid and protein adjustments.

Taken together, dietary cholesterol mainly by using the egg as a source can alter the lipid profile by increasing the markers in general. However, in assessing the actual biological impact this appears to be practically insignificant. Genetic factors can increase the cholesterol, LDL and triglycerides levels of individuals because of higher cholesterol intake, but nonetheless not quite alarming.
